# Variation in the association between socioeconomic status and breastfeeding practices by immigration status in Taiwan: a population based birth cohort study

**DOI:** 10.1186/s12884-015-0732-8

**Published:** 2015-11-16

**Authors:** Wen-chi Wu, Jennifer Chun-Li Wu, Tung-liang Chiang

**Affiliations:** Department of Health Healing and Health Marketing, School of Healthcare Management, Kainan University, No.1 Kainan Road, Luzhu Dist., Taoyuan City, 338 Taiwan; Department of Early Childhood and Family Education, College of Education, National Taipei University and Education, No.134, Sec. 2, Heping E. Road., Taipei, 106 Taiwan; Institute of Health Policy and Management, College of Public Health, National Taiwan University, Room 620, 6F, No. 17, Xuzhou Road., Taipei, 100 Taiwan

**Keywords:** Breastfeeding, Immigrants, Socioeconomic status, Education, Income

## Abstract

**Background:**

The relationship between socioeconomic status (SES) and breastfeeding has been extensively discussed in the literature. However, there is some evidence that this relationship can differ with immigration status. To date the majority of research investigating the relationships among SES, breastfeeding and immigration status has been conducted in Europe and the United States with a lack of similar research from Asia. Therefore, the aim of this study was to describe differences in breastfeeding practices between native-born Taiwanese mothers and immigrant mothers in Taiwan and to investigate any differences in the relationship between SES and breastfeeding practices by immigration status.

**Methods:**

Data analyzed came from the Taiwan Birth Cohort Study, the first longitudinal study of babies in Taiwan with a nationally representative sample born in 2005. In the present study, we included 21,217 mothers or primary caregivers who completed interview surveys when their child was 6 months old. Socioeconomic status was measured by the education level of mothers and fathers, and the couple’s monthly income. Data analysis involved multiple logistic regression. Control variables included residential area, mother’s employment status, age of the father and mother, and sex of the infant.

**Results:**

The proportion of immigrant mothers predominantly breastfeeding for 4 and for 6 months (Mainland Chinese mothers: 18.25 %, 36.29 %; Southeast Asian mothers: 10.71 %, 24.85 %) was significantly higher than that observed in their Taiwan-born counterparts (7.03 %, 16.22 %). Analysis stratified by immigration status showed that a higher level of parental education was associated with a greater likelihood of predominantly breastfeeding in Taiwanese mothers. However, no statistically significant relationship was observed between education and predominantly breastfeeding in immigrant mothers. Higher monthly income was also significantly associated with a greater likelihood of predominantly breastfeeding in Taiwanese mothers. However, there was no significant linear relationship between monthly income and predominantly breastfeeding in immigrant women.

**Conclusion:**

The relationship between SES and breastfeeding is different in immigrant mothers and native-born Taiwanese mothers. Taiwanese policy should continue to encourage breastfeeding in immigrant mothers. However, greater attention should be placed on Taiwanese mothers from a low SES background.

**Electronic supplementary material:**

The online version of this article (doi:10.1186/s12884-015-0732-8) contains supplementary material, which is available to authorized users.

## Background

The benefits of breastfeeding are well established. In addition to benefiting the baby (such as by preventing childhood diarrhea and respiratory infections [1]), breastfeeding also reduces the risk of breast cancer in mothers, delays the resumption of menses and accelerates the recovery of pre-pregnancy weight [2–5]. The World Health Organization (WHO) recommends exclusive breastfeeding until the infant is six months of age [4, 6, 7]. However, despite these clear benefits breastfeeding is still not universally adopted by all mothers. A WHO analysis of 108 member countries of the United Nations found that only 32.07 % of infants were exclusively breastfed until the age of 6 months [8]. Many factors influence whether or not a mother breastfeeds, with one of the most frequently considered factors being socioeconomic status (SES) [9].

SES is often measured by parental education level and household income. Research from a large number of different countries has found that high SES is associated with a greater likelihood of breastfeeding (e.g. Canada [10], New Zealand [11], Australia [12], and the United States [13–15]). One study from Taiwan has also found that high maternal education is associated with a greater likelihood of breastfeeding [16]. However, research from Sichuan province in China found that maternal education and household income were negatively associated with breastfeeding [17]. In Hong Kong, a positive relationship was observed between education and breastfeeding but a negative relationship was observed between income and breastfeeding [18]. In addition, a study that surveyed five Southeast Asian countries found that although low maternal education was associated with an increased risk of non-exclusive breastfeeding in Vietnamese and Cambodian mothers, high household wealth and living in a community with a higher percentage of wealthy households were associated with an increased risk of non-exclusive breastfeeding in Indonesian mothers [19]. These findings underline how the relationship between SES and breastfeeding can differ between countries.

Many studies have also discussed the relationship between immigration status and breastfeeding. The majority of these studies have found that immigrant mothers are more likely to breastfeed than their non-immigrant counterparts including in the United States [15], Canada [10] and European countries [20–22]. A Spanish study found that immigrant mothers (from Latin America, Eastern Europe, Maghreb or sub-Saharan Africa) had a higher breastfeeding initiation rate than native-born Spanish mothers. However, Chinese immigrant mothers were an exception with a breastfeeding initiation rate lower than that of their Spanish-born counterparts [20]. An Australian study in a Melbourne hospital found that the breastfeeding initiation rate of Vietnamese immigrant mothers was the lowest compared to that observed in Australian-born mothers and Turkish immigrant mothers [23]. Therefore, it is possible that the breastfeeding rates of Asian immigrants to Europe and the United States are lower than those observed in immigrants from other countries. However, there is currently a lack of research about breastfeeding practices in immigrants to Asian countries.

SES is related to breastfeeding behavior but it’s possible that this relationship is affected by immigration status. A US study used data from the National Survey of Children’s Health to examine the interaction between SES, immigration status and breastfeeding [24]. They found a six month breastfeeding rate of 42.5 % in US-born mothers of US-born infants with high parental education compared to a rate of 20.7 % in those with low parental education − a gap of 21.8 %. However, in immigrant couples with a US-born child, the difference in breastfeeding rates between those with high and low parental education was only 5.7 % (49.6–43.9 %). Findings were similar for household income. In US-born couples with a US-born child, the difference in breastfeeding rates between the richest and poorest families for breastfeeding at six months was 22.8 % (45.6–22.8 %). However, for immigrant couples with a US-born child, this difference in breastfeeding rates was only 9.1 % (53.3–44.2 %). A study from Belgium has also found no relationship between poverty level and breastfeeding in immigrants from Eastern Europe, outside of Europe, Turkey or Morocco [22]. However, in native-born Belgian mothers and immigrant mothers from Northern or Western Europe, poverty was associated with reduced breastfeeding. There is currently a lack of similar research on the differing relationship between SES and breastfeeding by immigration status in Asia.

Taiwan is similar to other East Asian countries (such as Korea and Japan), in the appearance of the phenomenon of international marriages at the end of the 20th century [25]. In 1998, international marriages represented 14.1 % of all registered marriages in Taiwan. The proportion of international marriages increased to a peak of 28.4 % in 2003, following which rates of international marriage have gradually declined [25]. In Taiwan, international marriages predominantly consist of foreign brides immigrating to Taiwan through marriage and in 2003 the ratio of foreign brides to foreign grooms was 8 to 1 [25]. In that same year, two thirds of foreign brides came from China (including Hong Kong and Macau). The other one third predominantly came from Southeast Asia (including Vietnam and Indonesia) [26]. In 2004 an estimated 13.19 % of births in Taiwan were to immigrant mothers, with one out of every eight children born to an immigrant mother [27]. Therefore, data from Taiwan could be useful in examining differences in breastfeeding practices between immigrant and native-born mothers in Asia.

We analyzed data from the Taiwan Birth Cohort Study (TBCS) [28] to examine the relationships between SES, immigration status and breastfeeding. TBCS is a nationally representative study that was established with a birth cohort in 2005. As this was right after the peak of international marriage migration to Taiwan, this data is well suited to examining differences in breastfeeding practices between Taiwanese and immigrant mothers. The aims of this study were to: 1. Describe any differences in breastfeeding practices between Taiwanese and immigrant mothers; 2. Examine the relationship between SES and breastfeeding; 3. Investigate differences in the relationship between SES and breastfeeding by immigration status.

## Methods

### Participants

Data were obtained from the Taiwan Birth Cohort Study (TBCS), the first longitudinal study in Taiwan where a nationally representative sample of 24,200 children were observed beginning from their birth in 2005. Using a life course approach, the TBCS aims to document the health and developmental profiles of Taiwanese children, investigate the influences of the social environment on children’s health, and examine the effect of early life events on health during adulthood [28]. Four waves of surveys have been administered to the cohort prior to them entering school at the ages of 6 months, 18 months, 3 years, and 5.5 years. Surveys were conducted through face-to-face interviews with mothers or primary caregivers after obtaining their informed consent, and a range of information regarding the social and physical environment was collected. For immigrant mothers, translated versions of selected assessment scales and terminology were provided during the interview to facilitate communication. Information about study purpose and procedures, sensitive questions, confidentiality, compensation, and the rights to refuse or quit the interview were all written on the informed consent form. Participants expressed their understanding of these issues before signed consent was obtained. The survey protocol and questionnaires of the Taiwan Birth Cohort Study as well as proposed research topics using the database including this study, have been approved by the IRB of the Bureau of Health Promotion, Department of Health and the Directorate-General of Budget, Accounting, and Statistics, Executive Yuan, ROC (No. 94-C3-0940005257). The consent form included the name of the ethics committee, description of the ethical issues, and the permission of publishing for academic purposes.

Initially, we included 21,248 women who completed the 6-month survey which equated to a response rate of 87.8 %. However, to achieve precise analysis results, we removed 31 mothers (0.015 %) who emigrated from countries other than China or those in Southeast Asia. The final analytical sample comprised 21,217 infants with the ages reported in the survey ranging from 165 to 195 days old.

### Measurements

#### Breastfeeding

As the TBCS lacked data on the provision of water to infants during the period of breastfeeding, we have used predominant breastfeeding rather than exclusive breastfeeding in the present study. The definition of predominant breastfeeding was adopted from a 2010 World Health Organization report [29] and indicates that the infant’s predominant source of nourishment has been breast milk (including milk expressed or from a wet nurse as the predominant source of nourishment). However, the infant may also have received liquids, (water and water-based drinks, fruit juice), ritual fluids and ORS, drops or syrups (vitamins, minerals and medicines).

Breastfeeding behavior was measured by the following questions in the TBCS. The first question to mothers was: “Did you ever breast feed your baby?” Those who responded yes were then asked to continue on to question two which was: “In the last six months, did you mainly breastfeed your baby and/or use baby formula? How long for?” There were four possible responses to question two: (1) Only breast milk; (2) Mainly breast milk that was supplemented with baby formula; (3) Mainly baby formula that was supplemented by breast milk; (4) Only baby formula. For each response, participants were asked to report the number of days or months that they breastfed/formula fed their baby for. The maximum total time frame for the combined four categories was 195 days or 6.5 months.

We grouped breastfeeding into three categories: ever breastfeeding, predominant breastfeeding (FBF) for four months, and predominant breastfeeding for six months. Ever breastfeeding refers to those mothers who answered yes to question one. Predominant breastfeeding for four months refers to those mothers who chose response one for question two (only breast milk) and reported a time period of more than 120 days. During this time period the infant was not given pureed fruit, pureed vegetables, rice-based products such as rice bran, rice cereal and rice flour, wheat-based products such as wheat cereal and wheat flour, or rice porridge. Predominant breastfeeding for six months refers to those mothers who chose response one for question two (only breast milk) and reported a time period of more than 180 days. They also did not feed their infant any of the above complementary foods.

#### Socioeconomic indicators

The education level of both parents and the couple’s monthly income were used as socioeconomic indicators. Education level was divided into 3 groups: 13 or more years, 10–12 years, and 9 or fewer years. Monthly income was measured as an ordinal variable with a total of five categories: New Taiwan (NT) dollars $100 000 (NT$1 ≈ US$0.03) or more; NT$70,000–NT$100,000; NT$50,000–NT$70,000; NT$30,000–NT$50,000; and NT$30,000or less. These income categories are generally representative of the five income groups in Taiwan in 2005 [30].

#### Immigration status

In this study immigration status was defined as being either an immigrant mother or a native-born Taiwanese mother. As the majority of immigrant mothers in Taiwan have come from Mainland China and Southeast Asia, we divided immigrant mothers in this study into the two broad groups of mothers from China and mothers from Southeast Asia.

#### Other background factors

We controlled for residential area, mother’s employment status, age of the father and mother, and sex of the infant. Residential area was categorized as rural (villages and townships) and urban areas (cities and municipalities). The employment status of the mother was defined as being either employed or unemployed. The ages of the mother and father were divided into two groups according to their 50th percentiles.

### Statistical analysis

Descriptive statistics were used to present the prevalence of breastfeeding among Taiwanese mothers and immigrant mothers. The associations between social determinants and breastfeeding rates were examined using the Chi-squared test. Multiple logistic regressions were used to investigate the effect of socioeconomic status on the breastfeeding practices of Taiwanese and immigrant mothers, and the odds ratios (OR) and 95 % confidence intervals (CIs) were presented. Statistical analysis was conducted using SAS 9.2 software.

## Results

### Differences in SES and background factors between Taiwanese and immigrant mothers

Table 1 shows the distribution of SES and other characteristics in mothers born in Taiwan, China and Southeast Asia. Out of the 21,217 participants in the study, 86.8 % were Taiwanese mothers, 4.5 % were Chinese mothers, and 8.67 % were Southeast Asian mothers. Taiwanese mothers had the highest proportion of mothers with 13 or more years of education (50.84 %), followed by mothers from China (11.2 %), and with the lowest proportion with this education level found in mothers from Southeast Asia (5.84 %). Similarly, education was also highest in partners of Taiwanese mothers with 50.65 % having 13 or more years of education compared to 27.85 % of partners of Chinese mothers and only 10.38 % of partners of Southeast Asian mothers. Taiwanese mothers had a similar education level to their partners, whereas immigrant mothers usually had a lower education level than their partners. The couple’s monthly income in native-born Taiwanese families (12.72 %, NT$100,000 or more; 8.75 %, NT$30,000 or less) was significantly higher than the income of families with a Chinese mother (2.93 %, NT$100,000 or more; 24.08 %, NT$30,000 or less) and the income of families with a Southeast Asian mother (0.82 %, NT$100,000 or more; 32.25 %, NT$30,000 or less).

**Table 1:** Distributions of socioeconomic status and control variables by immigration status

			Taiwanese mothers		Immigrant mothers				
	Total				China		Southeast Asia		*p*
	N	(%)	N	(%)	N	(%)	N	(%)	
Total	21217	(100.00)	18416	(100.00)	955	(100.00)	1839	(100.00)	
Socioeconomic status									
Mother’s education level									
1. ≧ 13 years	9564	(45.16)	9349	(50.84)	107	(11.20)	107	(5.84)	<.0001
2. 10 to 12 years	8476	(40.02)	7670	(41.71)	355	(37.17)	449	(24.52)	
3. ≦ 9 years	3139	(14.82)	1371	(7.46)	493	(51.62)	1275	(69.63)	
Missing = 41	38								
Father’s education level									
1. ≧ 13 years	9705	(46.12)	9250	(50.65)	264	(27.85)	190	(10.38)	<.0001
2. 10 to 12 years	8407	(39.95)	7109	(38.93)	430	(45.36)	866	(47.32)	
3. ≦ 9 years	2930	(13.92)	1902	(10.42)	254	(26.79)	774	(42.30)	
Missing = 175	175								
Parental monthly income									
1. ≧NT$100,000	2378	(11.25)	2335	(12.72)	28	(2.93)	15	(0.82)	<.0001
2. NT$70,000 ~ NT$100,000	4419	(20.90)	4328	(23.57)	47	(4.92)	42	(2.30)	
3. NT$50,000 ~ NT$70,000	5502	(26.02)	5086	(27.70)	173	(18.12)	242	(13.25)	
4. NT$30,000 ~ NT$50,000	6364	(30.10)	5003	(27.25)	477	(49.95)	884	(48.39)	
5. ≦ NT$30,000	2381	(11.73)	1607	(8.75)	230	(24.08)	644	(35.25)	
Missing = 73	73								
Control variables									
Residential area									
1. Rural	8981	(42.33)	7548	(40.99)	411	(42.86)	1019	(55.41)	<.0001
2. Urban	12236	(57.67)	10868	(59.01)	548	(57.14)	820	(44.59)	
Mother’s employment status									
1. Unemployed	8939	(42.17)	6843	(37.20)	755	(78.73)	1340	(72.91)	<.0001
2. Employed	12257	(57.83)	11553	(62.80)	204	(21.27)	498	(27.09)	
Missing = 21	21								
Mothers’ age									
1. age < = 50th percentile = 29	11873	(55.96)	9640	(52.35)	626	(65.28)	1604	(87.22)	<.0001
2. age > 50th percentile = 29	9344	(44.04)	8776	(47.65)	333	(34.72)	235	(12.78)	
Fathers’ age									
1. age < 50th percentile = 32	10710	(50.76)	10036	(54.85)	225	(23.49)	447	(24.31)	<.0001
2. age > 50th percentile = 32	10388	(49.24)	8262	(45.15)	733	(76.51)	1392	(75.69)	
Missing = 119	119								
Child’s Sex									
1. Girl	10091	(47.56)	8755	(47.54)	449	(46.82)	885	(48.12)	-
2. Boy	11126	(52.44)	9661	(52.46)	510	(53.18)	954	(51.88)	
Average years of living in Taiwan						3.4		3.3	-
Missing = 3									

The majority of Taiwanese and Chinese mothers lived in urban areas (59.0 %, 57.14 %), however, mothers from Southeast Asia were more likely to live in rural areas (55.41 %). More than sixty percent of Taiwanese mothers were employed when their infants were 6 months old (62.8 %). By contrast, immigrant mothers were more likely to be unemployed (78.73 % of Chinese mothers, 72.91 % of Southeast Asian mothers). Immigrant mothers were younger than their Taiwanese counterparts. However, the partners of immigrant mothers were older than the partners of Taiwanese mothers. The sex ratio of infants was not significantly different between Taiwanese and immigrant mothers. The average years lived in Taiwan was not different between Chinese (3.4 years) and Southeast Asian mothers (3.3 years). In summary, compared with Taiwanese mothers, immigrant mothers were younger, less educated and less wealthy, and were more likely to be living in rural areas, unemployed and married to older, less educated husbands.

### Differences in breastfeeding rates between Taiwanese and immigrant mothers

In this study 82.12 % of the mothers reported that they had ever breastfed in the last six months, 17.87 % of the mothers reported that they predominantly breastfed their child from birth to four months of age, and 7.95 % of the mothers reported that they predominantly breastfed their child from birth to six months of age (Table 2). The percentage of Southeast Asian mothers ever breastfeeding (76.94 %) was lower than that of Taiwanese and Chinese mothers (82.63 %; 82.27 %). The percentages of immigrant mothers predominantly breastfeeding for four months (36.29 % for Chinese mothers; 24.85 % for Southeast Asian mothers) was higher than that of Taiwanese mothers (16.2 %). The percentages of immigrant mothers predominantly breastfeeding for six months (18.25 % for Chinese mothers; 10.71 % for Southeast Asian mothers) was also higher than that of Taiwanese mothers (7.03 %).

**Table 2:** Distribution of breastfeeding practices by immigration status

			Taiwanese mothers		Immigrant mothers				
	Total				China		Southeast Asia		*p*
	N	(%)	N	(%)	N	(%)	N	(%)	
Total	21217	(100.00)	18416	(100.00)	959	(100.00)	1839	(100.00)	
Breastfeeding practices									
Ever breastfeeding^bc^									
Yes	17424	(82.12)	15218	(82.63)	789	(82.27)	1415	(76.94)	<.0001
No	3793	(17.88)	3198	(17.37)	170	(17.73)	424	(23.06)	
Predominant breastfeeding continued to fourth month^abc^									
Yes	3792	(17.87)	2987	(16.22)	348	(36.29)	457	(24.85)	<.0001
No	17425	(82.13)	15429	(83.78)	611	(63.71)	1382	(75.15)	
Predominant breastfeeding continued to sixth month^abc^									
Yes	1666	(7.85)	1294	(7.03)	175	(18.25)	197	(10.71)	<.0001
No	19551	(92.15)	17122	(92.97)	784	(81.75)	1642	(89.29)	

^a^Significant difference at 0.05 level between Taiwan-born and China-born mothers

^b^Significant difference at 0.05 level between China-born and Southeast Asia-born mothers

^c^Significant difference at 0.05 level between Taiwan-born and Southeast Asia-born mothers

### Associations between SES and breastfeeding practices

Table 3 shows the associations among socioeconomic status and breastfeeding practices after controlling for residential area, employment status, age of the mother, age of the father, and sex of the child. Mothers with higher education were more likely to ever breastfeed as well as predominantly breastfeed for four months or six months. Mothers with partners who had more than 13 years of education were also more likely to ever breastfeed, predominantly breastfeed for four months and predominantly breastfeed for six months compared to mothers with partners who had less than 9 years of education. The relationship between the couple’s monthly income and breastfeeding did not reach statistical significance. However, the magnitude of the association was large and demonstrated a U shaped association. Breastfeeding rates were highest in those with the lowest and highest parental monthly incomes. Immigrant mothers remained more likely to breastfeed than Taiwanese mothers even after controlling for other factors and there was no change in the association between immigration status and breastfeeding in the adjusted compared to the unadjusted analysis (Table 1). We also tested for interactions between immigration status and mother’s education, father’s education and parental monthly income (Additional file 1) and found statistically significant (*p* < 0.05) interactions between immigration status and mother’s education and parental monthly income. Therefore, the most appropriate way to answer our study aims was to stratify the analysis by immigration status.

**Table 3:** The associations among SES, immigration status, and breastfeeding practices

	Ever breastfeeding			Predominant breastfeeding continued to fourth month			Predominant breastfeeding continued to sixth month		
	Rate	OR	(95 % CI)	Rate	OR	(95 % CI)	Rate	OR	(95 % CI)
Socioeconomic status									
Mother’s education									
1. ≧ 13 years	90.16	2.74	(2.38–3.16)^c^	21.30	2.16	(1.85–2.52)^c^	9.20	2.00	(1.62–2.47)^c^
2. 10 to 12 years	76.95	1.31	(1.17–1.50)^c^	13.67	1.20	(1.05–1.37)^a^	6.02	1.13	(0.94–1.36)
3. ≦ 9 years	71.84	1.00		18.89	1.00		8.73	1.00	
Father’s education									
1. ≧ 13 years	89.25	2.13	(1.88–2.43)^c^	21.66	1.62	(1.41–1.87)^c^	9.50	1.74	(1.43–2.12)^c^
2. 10 to 12 years	78.58	1.43	(1.29–1.58)^c^	14.43	1.10	(0.97–1.25)	6.35	1.14	(0.96–1.37)
3. ≦ 9 years	69.76	1.00		15.67	1.00		6.86	1.00	
Parental monthly income									
1. ≧ NT$100,000	90.03	1.32	(1.10–1.60)^a^	21.91	1.24	(1.05–1.48)^a^	8.96	0.97	(0.77–1.23)
2. NT$70,000 ~ NT$100,000	87.28	1.10	(0.94–1.28)	17.20	1.04	(0.89–1.22)	6.92	0.83	(0.67–1.03)
3. NT$50,000 ~ NT$70,000	81.42	0.91	(0.79–1.03)	15.50	0.95	(0.83–1.10)	6.32	0.76	(0.62–0.92)^b^
4. NT$30,000 ~ NT$50,000	78.39	0.90	(0.83–0.97)^a^	18.42	0.96	(0.89–1.03)	8.69	0.87	(0.73–1.02)^a^
5. ≦ NT$30,000	76.74	1.00		19.27	1.00		9.75	1.00	
Immigration status									
1. From China	82.27	1.60	(1.33–1.93)^c^	36.29	3.34	(2.84–3.91)^c^	18.25	2.89	(2.36–3.54)^c^
2. From Southeast Asia	76.94	1.39	(1.21–1.61)^c^	24.85	2.37	(2.04–2.76)^c^	10.71	1.89	(1.54–2.32)^c^
3. Taiwanese	82.63	1.00		16.22	1.00		7.03	1.00	

Note 1. ^a^: *p* < .05, ^b^: *p* < .01, ^c^: *p* < .001

Note 2. These models were controlled for the residential area, employment status, age of the mother, age of the father, and sex of the child

### Associations between SES and breastfeeding practices stratified by immigration status

Table 4 shows the associations between SES and breastfeeding practices stratified by immigration status after controlling for other background factors. No relationship was observed between mother’s education level and breastfeeding in mothers from China or Southeast Asia. However, Taiwanese women with higher education were more likely to breastfeed (including ever breastfeeding, predominant breastfeeding for four months and predominant breastfeeding for six months). Higher education in the partners of Chinese mothers was associated with a greater likelihood of ever breastfeeding. Higher education in partners of Southeast Asian mothers was also associated with a greater likelihood of ever breastfeeding. In Taiwanese mothers, higher education of partners was associated with a greater likelihood of all three categories of breastfeeding. There was no clear linear trend in the relationship between parental monthly income and breastfeeding in immigrant mothers. However, in Taiwanese mothers higher parental income was associated with a greater likelihood of all three categories of breastfeeding. Overall the lowest rate of breastfeeding was in Taiwanese mothers with an education level of less than nine years (3.43 %).

**Table 4:** The association between SES and breastfeeding practices by immigration status

Immigration status	Ever breastfeeding			Predominant breastfeeding continued to fourth month			Predominant breastfeeding continued to sixth month		
Socioeconomic status	Rate	OR	(95 % CI)	Rate	OR	(95 % CI)	Rate	OR	(95 % CI)
Mothers from China	(Yes = 774/No = 169)			(Yes = 345/No = 598)			(Yes = 172/No = 771)		
Mother’s education									
1. ≧ 13 years	85.05	0.98	(0.53–1.84)	34.58	0.99	(0.61–1.59)	17.76	1.06	(0.58–1.91)
2. 10 to 12 years	83.38	1.01	(0.69–1.48)	40.85	1.38	(1.02–1.87)^a^	18.59	1.15	(0.79–1.68)
3. ≦ 9 years	80.73	1.00		33.67	1.00		18.26	1.00	
Father’s education									
1. ≧ 13 years	88.26	2.45	(1.46–4.10)^c^	40.15	1.44	(0.96–2.15)	18.18	1.40	(0.84–2.33)
2. 10 to 12 years	82.79	1.59	(1.07–2.36)^a^	36.98	1.26	(0.89–1.78)	20.00	1.48	(0.96–2.29)
3. ≦ 9 years	74.80	1.00		31.50	1.00		14.96	1.00	
Parental monthly income									
1. ≧ NT$100,000	92.86	1.46	(0.32–6.79)	25.00	0.65	(0.25–1.70)	10.71	0.38	(0.08–1.71)
2. NT$70,000 ~ NT$100,000	80.85	0.59	(0.25–1.39)	38.30	1.17	(0.59–2.32)	17.02	0.80	(0.34–1.89)
3. NT$50,000 ~ NT$70,000	79.19	0.59	(0.34–1.01)	34.10	1.09	(0.70–1.69)	16.18	0.79	(0.46–1.36)
4. NT$30,000 ~ NT$50,000	81.34	0.67	(0.43–1.05)	38.78	1.24	(0.89–1.75)	18.03	0.82	(0.55–1.24)
5. ≦ NT$30,000	85.22	1.00		34.35	1.00		21.74	1.00	
Mothers from Southeast Asia	(Yes = 1398/No = 414)			(Yes = 451/No = 1361)			(Yes = 194/No = 1618)		
Mother’s education									
1. ≧ 13 years	74.77	0.94	(0.56–1.57)	25.23	1.34	(0.79–2.26)	10.28	1.10	(0.52–2.33)
2. 10 to 12 years	78.17	1.10	(0.84–1.43)	25.17	1.10	(0.85–1.43)	10.69	1.09	(0.76–1.56)
3. ≦ 9 years	76.78	1.00		24.78	1.00		10.75	1.00	
Father’s education									
1. ≧ 13 years	80.00	1.74	(1.14–2.66)^a^	25.26	1.13	(0.76–1.69)	13.16	1.62	(0.95–2.75)
2. 10 to 12 years	80.60	1.60	(1.27–2.03)^c^	25.64	1.12	(0.89–1.42)	11.09	1.24	(0.89–1.72)
3. ≦ 9 years	72.35	1.00		23.90	1.00		9.69	1.00	
Parental monthly income									
1. ≧ NT$100,000	80.00	1.23	(0.33–4.54)	33.33	1.36	(0.43–4.29)	26.67	3.18	(0.90–11.31)
2. NT$70,000 ~ NT$100,000	71.43	0.70	(0.33–1.43)	11.90	0.35	(0.13–0.94)^a^	7.14	0.61	(0.18–2.01)
3. NT$50,000 ~ NT$70,000	69.01	0.64	(0.45–0.91)^a^	21.07	0.78	(0.54–1.13)	7.44	0.66	(0.38–1.15)
4. NT$30,000 ~ NT$50,000	78.28	1.01	(0.78–1.30)	22.85	0.71	(0.56–0.89)^b^	9.50	0.68	(0.49–0.94)^a^
5. ≦ NT$30,000	78.57	1.00		29.66	1.00		13.51	1.00	
Taiwanese mothers	(Yes = 15067/No = 3114)			(Yes = 2969/No = 15212)			(Yes = 1286/No = 16895)		
Mother’s education									
1. ≧ 13 years	90.39	3.25	(2.78–3.82)^c^	21.10	2.75	(2.19–3.45)^c^	9.09	2.89	(2.07–4.03)^c^
2. 10 to 12 years	76.58	1.52	(1.33–1.74)^c^	11.75	1.45	(1.21–1.86)^c^	5.16	1.61	(1.17–2.22)^b^
3. ≦ 9 years	64.04	1.00		8.10	1.00		3.43	1.00	
Father’s education									
1. ≧ 13 years	89.47	2.03	(1.76–2.34)^c^	21.06	1.63	(1.36–1.92)^c^	9.18	1.64	(1.26–2.12)^c^
2. 10 to 12 years	78.08	1.36	(1.20–1.53)^c^	11.70	1.07	(0.90–1.27)	4.95	1.01	(0.79–1.30)
3. ≦ 9 years	68.03	1.00		10.20	1.00		4.63	1.00	
Parental monthly income									
1. ≧ NT$100,000	90.06	1.35	(1.10–1.66)^a^	21.80	1.39	(1.13–1.70)^b^	8.82	1.04	(0.78–1.38)
2. NT$70,000 ~ NT$100,000	87.52	1.14	(0.96–1.35)	17.03	1.16	(0.96–1.41)	6.82	0.90	(0.69–1.78)
3. NT$50,000 ~ NT$70,000	82.09	0.97	(0.84–1.13)	14.61	1.07	(0.89–1.28)	5.94	0.84	(0.65–1.07)
4. NT$30,000 ~ NT$50,000	78.13	0.91	(0.79–1.05)	15.69	1.09	(0.91–1.29)	7.66	1.02	(0.81–1.84)
5. ≦ NT$30,000	74.80	1.00		12.94	1.00		6.53	1.00	

Note 1. ^a^: *p* < .05, ^b^: *p* < .01, ^c^: *p* < .001

Note 2. These models controlled for the residential area, employment status, age of the mother, age of the father, and sex of the child

## Discussion

We found that the rate of ever breastfeeding was slightly lower in Southeast Asian mothers compared to Chinese and Taiwanese mothers. However, the rate of predominant breastfeeding for four or six months was significantly higher in immigrant mothers compared to Taiwanese mothers. Stratified analyses demonstrated that the relationship between SES and breastfeeding differed between Taiwanese and immigrant mothers. For Taiwanese mothers, a higher education level in themselves or their partners was associated with a greater likelihood of breastfeeding (including ever breastfeeding and predominant breastfeeding for 4 and 6 months). However, in immigrant mothers no statistically significant relationship was observed between mother’s education level and breastfeeding and father’s education level only had a positive association with ever breastfeeding. In terms of parental monthly income, a positive association was only observed between income and breastfeeding in Taiwanese mothers. No significant association between income and breastfeeding was observed in immigrant mothers.

### Immigrant mothers are more likely to continue breastfeeding than non-immigrant mothers

The predominant breastfeeding rate for four months and for six months was higher in immigrant mothers compared to Taiwanese mothers. This finding supports previous research from Europe and the United States [10, 15, 20–22]. The majority of the immigrant mothers in this study had moved to Taiwan from Vietnam (49.7 %), China (33.9 %), Indonesia (8.4 %), and Cambodia (2.9 %). According to a World Bank report [31], 3-month exclusive breastfeeding rates are 19.5 % in Vietnam, 53.6 % in Indonesia, and 32.5 % for the whole of East Asia. In China, a survey conducted by the United Nations Children’s Fund (UNICEF) reported that the rate of exclusive breastfeeding for 6 months was 28 % [32]. These statistics show that the majority of immigrant mothers in Taiwan came from countries with a higher prevalence of breastfeeding than Taiwan (7.9 %). It is likely that these women have brought with them culture-related practices relating to breastfeeding from their original countries to the new host country.

### Associations between SES and breastfeeding practices varied between Taiwanese and immigrant mothers

Our results indicate that a higher parental education level is beneficial for breastfeeding practices among Taiwanese mothers, but no statistically significant beneficial effect of higher education on breastfeeding was observed in immigrant mothers. The gradient of breastfeeding rates across educational levels in Taiwanese mothers is consistent with the gradient observed in other developed countries [12, 33]. Nonetheless, we still found that the breastfeeding rates in highly educated immigrant mothers were similar to the rates in less educated immigrant mothers.

One possible explanation could be derived from the breastfeeding transition perspective proposed by Cattaneo [34] which comprises 3 phases: (1) the initial phase: high prevalence and duration of breastfeeding without socioeconomic disparity; (2) the transformation phase: the prevalence and duration of breastfeeding begins to decline first among the urban elite, and then among the rural and poor; and (3) the resurgence phase: an inverse trend of increasing prevalence and duration of breastfeeding that occurs first among the urban elite, and then among the rural and poor.

According to this perspective and related literature [35–38], we propose that the low and middle income countries where the mothers emigrated from may be in the transformation phase (highest SES had lowest BF rate), whereas based on our results Taiwan is in the resurgence phase (highest SES had highest BF rate). Therefore, the diminished socioeconomic disparity among immigrant mothers could be due to their moving to a country in a later phase of breastfeeding transition where they may later undergo the process of transition from the transformation phase to the resurgence phase. The comparable breastfeeding rates across education levels could imply that this transition process has not been completed in these immigrant women; otherwise, breastfeeding rates would be highest among immigrant mothers with the highest education. Therefore, our results show that not only is the rate of breastfeeding higher in immigrant mothers compared to Taiwanese mothers but that it is also less susceptible to the impact of SES. Despite higher current levels of breastfeeding, due to the overwhelming benefits of breastfeeding it is important for the health of both these immigrant women and their children that these high levels of breastfeeding are maintained. Therefore, policy measures that ensure equitable access to maternal and child health services regardless of immigration background should be in place to support the breastfeeding practices of immigrant mothers.

At the present time in Taiwan all pregnant women receive pregnancy-related health education. Therefore, once immigrant women become pregnant they will also receive this health education in the form of antenatal and postnatal classes [39, 40]. In 1992 the Department of Health launched a breastfeeding promotion project that restricted the promotional and advertising activities of milk companies, provided public breastfeeding education, and encouraged businesses to establish breastfeeding rooms in the workplace. In addition, in 2001 the Health Promotion Administration in Taiwan implemented a national mother- baby-friendly hospital certification system based on the WHO’s UNICEF program of building breastfeeding friendly environments for mothers. The health education program for pregnant women is provided to immigrant mothers in their native language and is provided free of charge. Such an approach should ensure equity of access to this program by immigrant mothers and should help support the positive breastfeeding practices of these mothers.

### Parental education is more strongly related to breastfeeding practices than income

In Taiwanese mothers, a higher education level in mothers or their partners was associated with a greater likelihood of breastfeeding. However, unlike education breastfeeding rates did not significantly differ across parental income levels. A possible explanation may lie in the fundamental meanings of income and education. Income mainly involves material resources while education reflects a pile of non-fiscal resources such as knowledge, problem solving skills, social elite connections and the power of influence [41]. Breastfeeding is recognized as a natural practice that needs no additional equipment or money. However, it does require updated knowledge about breastfeeding, accessible social network connections, determination to conduct breastfeeding and problem solving skills. From this viewpoint, income may play a less important role in breastfeeding than education [42].

### Limitations

Several limitations in this study must be mentioned. First, immigrant mothers may represent a group that are different from women in their country of origin, such as differences due to the healthy immigrant effect [43]. Because this was a natural experiment, selection bias is difficult to dismiss. Second, psychosocial factors related to the level of cultural acceptance should have been considered, such as the level of perceived mother-baby friendliness from the environment, perceived family attitudes toward breastfeeding, and engagement in parental activities. Future research should include additional factors related to breastfeeding practices to clarify the mechanisms that link socioeconomic status to breastfeeding practices. Third, TBCS did not collect information about the provision of water to infants during the period of breastfeeding and as a result in this study we could only measure predominant breastfeeding rather than exclusive breastfeeding.

## Conclusion

This study found that the rates of predominant breastfeeding for 4 and 6 months were higher in immigrant mothers compared to their native-born Taiwanese counterparts. In addition, no significant gradient effects of parental education level on breastfeeding were observed in immigrant mothers as there were in Taiwanese mothers. In Taiwanese mothers the associations between SES and breastfeeding practices was mainly explained by variation in parental education rather than income level. Therefore, education interventions designed to increase knowledge of breastfeeding could be effective in encouraging breastfeeding practices. In addition, particular attention may be needed for less educated Taiwanese mothers.

## Additional file

Additional file 1:
**Interactions between immigration status and SES.** (DOCX 19 kb)
