# Supplementary material for: Variation in the association between socioeconomic status and breastfeeding practices by immigration status in Taiwan: a population based birth cohort study
Source: BMC Pregnancy Childbirth. 2015 Nov 16;15:298. doi: 10.1186/s12884-015-0732-8 (PMC4647632; doi:10.1186/s12884-015-0732-8)
Supplement: Additional file 1: — Interactions between immigration status and SES. (DOCX 19 kb) [file 12884_2015_732_MOESM1_ESM.docx]

Additional file 1. Interactions between immigration status and SES

|  | Ever breastfeeding | | | Predominant breastfeeding continued to fourth month | | | Predominant breastfeeding continued to sixth month | | |
| --- | --- | --- | --- | --- | --- | --- | --- | --- | --- |
|  | Beta | S.E. | P-value | Beta | S.E. | P-value | Beta | S.E. | P-value |
| Main effects |  |  |  |  |  |  |  |  |  |
| Immigration status |  |  |  |  |  |  |  |  |  |
| 1. From China / 3. Taiwanese | 0.94 | 0.23 | **<.0001** | 1.43 | 0.22 | **<.0001** | 1.56 | 0.29 | **<.0001** |
| 2. From Southeast Asia / 3. Taiwanese | 0.56 | 0.14 | **<.0001** | 1.47 | 0.17 | **<.0001** | 1.22 | 0.24 | **<.0001** |
| Socioeconomic status |  |  |  |  |  |  |  |  |  |
| Mother's education |  |  |  |  |  |  |  |  |  |
| 1. ≧ 13 years / 3. ≦ 9 years | 1.18 | 0.08 | **<.0001** | 1.01 | 0.12 | **<.0001** | 1.07 | 0.17 | **<.0001** |
| 2. 10 to 12 years / 3. ≦ 9 years | 0.42 | 0.07 | **<.0001** | 0.41 | 0.11 | **0.000** | 0.48 | 0.16 | **0.004** |
| Father's education |  |  |  |  |  |  |  |  |  |
| 1. ≧ 13 years / 3. ≦ 9 years | 0.71 | 0.07 | **<.0001** | 0.49 | 0.09 | **<.0001** | 0.50 | 0.13 | **0.000** |
| 2. 10 to 12 years / 3. ≦ 9 years | 0.31 | 0.06 | **<.0001** | 0.07 | 0.09 | 0.462 | 0.02 | 0.13 | 0.906 |
| Parental monthly income |  |  |  |  |  |  |  |  |  |
| 1. ≧ NT$100,000 / 5. ≦ NT$30,000 | 0.31 | 0.10 | **0.002** | 0.32 | 0.10 | **0.002** | 0.08 | 0.14 | 0.567 |
| 2. NT$70,000 ~ NT$100,000 / 5. ≦ NT$30,000 | 0.14 | 0.09 | 0.094 | 0.15 | 0.10 | 0.127 | -0.07 | 0.13 | 0.602 |
| 3. NT$50,000 ~ NT$70,000 / 5. ≦ NT$30,000 | -0.02 | 0.08 | 0.814 | 0.07 | 0.09 | 0.481 | -0.16 | 0.13 | 0.215 |
| 4. NT$30,000 ~ NT$50,000 / 5. ≦ NT$30,000 | -0.09 | 0.07 | 0.190 | 0.08 | 0.09 | 0.358 | 0.03 | 0.12 | 0.831 |
| Interaction terms |  |  |  |  |  |  |  |  |  |
| Immigration status * Mother's education |  |  |  |  |  |  |  |  |  |
| 1. From China * 1. ≧ 13 years | -1.18 | 0.33 | **0.000** | -1.03 | 0.27 | **0.000** | -1.02 | 0.35 | **0.003** |
| 1. From China * 2. 10 to 12 years | -0.40 | 0.21 | 0.053 | -0.10 | 0.19 | 0.600 | -0.37 | 0.25 | 0.139 |
| 2. From Southeast Asia * 1. ≧ 13 years | -1.35 | 0.27 | **<.0001** | -0.80 | 0.28 | **0.004** | -1.12 | 0.41 | **0.006** |
| 2. From Southeast Asia * 2. 10 to 12 years | -0.36 | 0.15 | **0.018** | -0.32 | 0.17 | 0.058 | -0.43 | 0.24 | 0.080 |
| Immigration status * Father's education |  |  |  |  |  |  |  |  |  |
| 1. From China * 1. ≧ 13 years | 0.21 | 0.27 | 0.444 | -0.17 | 0.22 | 0.446 | -0.21 | 0.29 | 0.472 |
| 1. From China * 2. 10 to 12 years | 0.17 | 0.21 | 0.416 | 0.14 | 0.20 | 0.466 | 0.36 | 0.25 | 0.151 |
| 2. From Southeast Asia * 1. ≧ 13 years | -0.17 | 0.23 | 0.462 | -0.37 | 0.22 | 0.100 | -0.05 | 0.30 | 0.860 |
| 2. From Southeast Asia * 2. 10 to 12 years | 0.17 | 0.13 | 0.208 | 0.06 | 0.15 | 0.707 | 0.19 | 0.21 | 0.367 |
| Immigration status * Parental monthly income |  |  |  |  |  |  |  |  |  |
| 1. From China * 1. ≧ NT$100,000 | 0.06 | 0.78 | 0.935 | -0.73 | 0.49 | 0.140 | -1.14 | 0.78 | 0.145 |
| 1. From China * 2. NT$70,000 ~ NT$100,000 | -0.69 | 0.44 | 0.115 | 0.03 | 0.36 | 0.936 | -0.15 | 0.46 | 0.745 |
| 1. From China * 3. NT$50,000 ~ NT$70,000 | -0.55 | 0.28 | **0.054** | 0.04 | 0.24 | 0.854 | -0.04 | 0.30 | 0.883 |
| 1. From China * 4. NT$30,000 ~ NT$50,000 | -0.29 | 0.24 | 0.219 | 0.12 | 0.19 | 0.534 | -0.22 | 0.24 | 0.361 |
| 2. From Southeast Asia * 1. ≧ NT$100,000 | -0.21 | 0.67 | 0.752 | -0.05 | 0.59 | 0.927 | 0.90 | 0.65 | 0.166 |
| 2. From Southeast Asia * 2. NT$70,000 ~ NT$100,000 | -0.55 | 0.37 | 0.137 | -1.19 | 0.51 | **0.019** | -0.50 | 0.64 | 0.435 |
| 2. From Southeast Asia * 3. NT$50,000 ~ NT$70,000 | -0.48 | 0.19 | **0.012** | -0.32 | 0.21 | 0.120 | -0.31 | 0.30 | 0.310 |
| 2. From Southeast Asia * 4. NT$30,000 ~ NT$50,000 | 0.09 | 0.15 | 0.554 | -0.43 | 0.15 | **0.004** | -0.43 | 0.20 | 0.037 |

Note 1. These models were controlled for residential area, employment status, age of the mother, age of the father, and sex of the child.

Note 2. S.E.: Standard Error.

Note 3. Bold type indicates p-value < 0.05.
